# Supplementary material for: A Likelihood Approach for Real-Time Calibration of Stochastic Compartmental Epidemic Models
Source: PLoS Comput Biol. 2017 Jan 17;13(1):e1005257. doi: 10.1371/journal.pcbi.1005257 (PMC5240920; doi:10.1371/journal.pcbi.1005257)
Supplement: S1 File — (TAR.GZ) [file pcbi.1005257.s014.tar.gz › HSPH_Online-SI-Revision/MSS10/n100-severe/n100-severe_NEW_table4.pdf]

1	R0	{1.65278, 9.06793, 0.1}	{1.67732, 9.44073, 0.}	{{1.67732, 9.44073, 0.}, {1.67732, 9.44073, 0.}}	{1.4847, 4.11118, 100.}	inf	4608.	5329.31	{5250.32, 5399.81}	15.6535	0.0631284	newly	3152.	3483.34	{3410., 3557.}	10.5121	0.0433554	Reff	0.666979	0.664783	{0.663275, 0.66608}	0.331513	ar	66342.	68244.	{67986., 68476.}	2.86692
2	R0	{1.4321, 3.80644, 0.1}	{1.44762, 4.00571, 0.}	{{1.44762, 4.00571, 0.}, {1.44762, 4.00571, 0.}}	{1.08374, 5.23507, 100.}	inf	684.	808.579	{729.57, 881.792}	18.2132	0.0716735	newly	952.	1077.14	{958., 1183.}	13.2416	0.0529548	Reff	0.675178	0.680692	{0.679617, 0.681827}	0.816639	ar	54217.	54585.6	{54307., 54841.}	0.687515
3	R0	{1.68388, 4.76028, 0.1}	{1.64953, 4.30394, 2.}	{{1.64953, 4.30394, 2.}, {1.64953, 4.30394, 2.}}	{2.03981, 9.58634, 1900.}	inf	788.	625.127	{572.123, 668.971}	20.6692	0.101428	newly	834.	715.84	{658., 771.}	14.247	0.0676568	Reff	0.549854	0.541325	{0.540507, 0.542156}	1.5512	ar	68299.	67901.1	{67772., 68026.}	0.582541
4	R0	{1.66014, 6.95383, 1.}	{1.67405, 7.28757, 1.}	{{1.67405, 7.28757, 1.}, {1.67405, 7.28757, 1.}}	{0.837854, 4.79943, 0.}	inf	3183.	3703.88	{3625.07, 3785.64}	16.3645	0.0657652	newly	2735.	2972.84	{2895., 3054.}	8.69616	0.0361216	Reff	0.623814	0.620321	{0.618898, 0.621605}	0.559966	ar	66820.	67999.9	{67708., 68313.}	1.76585
5	R0	{1.69984, 11.2038, 1.}	{1.48963, 2.29225, 3.12}	{{1.48799, 2.26472, 3.}, {1.48799, 2.26472, 3.}}	{12.3663, 79.5404, 212.}	inf	5824.	967.533	{855.586, 1069.51}	83.3871	0.781474	newly	3233.	1827.2	{1633., 2047.}	43.4828	0.24946	Reff	0.680072	0.668313	{0.666347, 0.66996}	1.7291	ar	68462.	56962.5	{56569., 57314.}	16.7969
6	R0	{1.65876, 6.66217, 1.}	{1.67405, 7.28757, 1.}	{{1.67405, 7.28757, 1.}, {1.67405, 7.28757, 1.}}	{0.921746, 9.3874, 0.}	inf	3387.	3823.13	{3741.68, 3919.24}	12.8766	0.052522	newly	2924.	3071.5	{2984., 3161.}	5.05951	0.0213359	Reff	0.632386	0.630914	{0.629085, 0.632259}	0.248907	ar	67105.	67677.2	{67430., 67919.}	0.852723
7	R0	{1.69966, 5.89996, 0.1}	{1.74092, 5.83416, 0.}	{{1.74092, 5.83416, 0.}, {1.74092, 5.83416, 0.}}	{2.42735, 1.11526, 100.}	inf	2191.	2645.56	{2571.89, 2730.77}	20.7467	0.0817423	newly	2014.	2475.05	{2383., 2568.}	22.8923	0.0893475	Reff	0.564848	0.570645	{0.569143, 0.571898}	1.02635	ar	69396.	70332.1	{70091., 70567.}	1.3489
8	R0	{1.53428, 6.3734, 0.1}	{1.47651, 5.4396, 3.}	{{1.47651, 5.4396, 3.}, {1.47651, 5.4396, 3.}}	{3.76532, 14.6515, 2900.}	inf	2878.	1962.11	{1864.44, 2052.1}	31.8238	0.166695	newly	2677.	2070.84	{1953., 2183.}	22.6433	0.111861	Reff	0.700629	0.687771	{0.686393, 0.689171}	1.83517	ar	59855.	57172.	{56851., 57500.}	4.48245
9	R0	{1.58939, 3.48608, 0.1}	{1.54098, 3.31039, 5.}	{{1.54098, 3.31039, 5.}, {1.54098, 3.31039, 5.}}	{3.04587, 5.03976, 4900.}	inf	666.	615.434	{523.746, 683.531}	9.31543	0.0434211	newly	876.	866.77	{756., 964.}	8.11301	0.0357596	Reff	0.606066	0.588385	{0.587245, 0.589665}	2.9173	ar	62575.	62677.2	{62470., 62874.}	0.241694
10	R0	{1.58528, 5.5593, 0.1}	{1.59034, 5.09935, 4.}	{{1.59034, 5.09935, 4.}, {1.59034, 5.09935, 4.}}	{0.319359, 8.27355, 3900.}	inf	2018.	1781.9	{1682., 1903.95}	11.6999	0.0544708	newly	2025.	1923.23	{1803., 2051.}	5.79802	0.0262237	Reff	0.636823	0.642611	{0.640654, 0.644092}	0.908984	ar	63039.	62473.1	{62161., 62727.}	0.897667
11	R0	{1.58305, 4.62721, 0.1}	{1.59034, 5.09935, 4.}	{{1.59034, 5.09935, 4.}, {1.59034, 5.09935, 4.}}	{0.460676, 10.2035, 3900.}	inf	1344.	1641.07	{1554.66, 1717.59}	22.1037	0.0863865	newly	1559.	1742.48	{1648., 1834.}	11.8191	0.0481419	Reff	0.61568	0.613792	{0.612541, 0.615087}	0.312922	ar	63222.	63785.9	{63545., 64018.}	0.891905
12	R0	{1.70234, 9.34626, 0.1}	{1.75054, 10.7687, 3.34}	{{1.62191, 8.12461, 2.}, {1.81389, 12.0711, 4.}}	{5.94968, 23.8466, 3240.}	inf	5033.	5985.03	{3779.94, 7123.79}	35.1302	0.139007	newly	3340.	3419.09	{2778., 3768.}	12.8817	0.056226	Reff	0.662312	0.663012	{0.650401, 0.669814}	1.22561	ar	68213.	70389.7	{65519., 72973.}	5.66662
13	R0	{1.68556, 9.74954, 1.}	{1.67732, 9.44073, 0.}	{{1.67732, 9.44073, 0.}, {1.67732, 9.44073, 0.}}	{0.488924, 3.16745, 100.}	inf	4862.	4893.76	{4799.45, 4981.41}	1.23841	0.00534722	newly	3072.	3180.2	{3102., 3259.}	3.59961	0.015288	Reff	0.650053	0.646342	{0.644861, 0.647852}	0.570865	ar	68426.	68402.2	{68093., 68685.}	0.264446
14	R0	{1.59876, 9.78897, 1.}	{1.56097, 8.77207, 2.}	{{1.56097, 8.77207, 2.}, {1.56097, 8.77207, 2.}}	{2.36362, 10.3883, 100.}	inf	5531.	5704.53	{5599.13, 5810.76}	3.14853	0.013422	newly	3640.	4092.17	{4014., 4184.}	12.4223	0.0507933	Reff	0.761314	0.74061	{0.738942, 0.742234}	2.71941	ar	63553.	63274.5	{62898., 63697.}	0.540069
15	R0	{1.43577, 5.12819, 1.}	{1.41571, 4.94504, 2.}	{{1.41571, 4.94504, 2.}, {1.41571, 4.94504, 2.}}	{1.39688, 3.57152, 100.}	inf	2030.	1836.32	{1729.64, 1941.9}	9.54094	0.0440227	newly	2303.	2150.11	{2012., 2285.}	6.87929	0.0313793	Reff	0.736378	0.728833	{0.727339, 0.730341}	1.02452	ar	53259.	52751.8	{52290., 53122.}	0.989711
16	R0	{1.58866, 8.73191, 1.}	{1.61188, 8.7843, 3.}	{{1.61188, 8.7843, 3.}, {1.61188, 8.7843, 3.}}	{1.46151, 0.599927, 200.}	inf	5612.	4936.14	{4838.43, 5027.34}	12.0432	0.0557789	newly	4036.	3539.92	{3454., 3635.}	12.2914	0.0570408	Reff	0.723984	0.745459	{0.743973, 0.747023}	2.96625	ar	64349.	63509.8	{63161., 63819.}	1.30409
17	R0	{1.43012, 3.8163, 0.1}	{1.4365, 3.42532, 3.76}	{{1.43579, 3.38828, 4.}, {1.43579, 3.38828, 4.}}	{0.445845, 10.8405, 3672.}	inf	1091.	980.963	{860.951, 1098.37}	11.4376	0.0537898	newly	1516.	1502.51	{1314., 1660.}	7.35686	0.0323967	Reff	0.70422	0.708939	{0.707186, 0.710552}	0.670092	ar	53353.	52858.9	{52388., 53323.}	0.977958
18	R0	{1.47965, 5.16472, 0.1}	{1.47651, 5.4396, 3.}	{{1.47651, 5.4396, 3.}, {1.47651, 5.4396, 3.}}	{0.212245, 5.3223, 2900.}	inf	1756.	2025.31	{1960.33, 2093.79}	15.3368	0.0617906	newly	1958.	2154.64	{2067., 2243.}	10.0429	0.0413443	Reff	0.705823	0.700348	{0.699331, 0.701297}	0.775622	ar	56086.	56649.8	{56288., 56958.}	1.01845
19	R0	{1.57191, 5.2314, 0.1}	{1.59034, 5.09935, 4.}	{{1.59034, 5.09935, 4.}, {1.59034, 5.09935, 4.}}	{1.17263, 2.52421, 3900.}	inf	1293.	1235.51	{1158.09, 1301.26}	5.02833	0.0226188	newly	1298.	1318.92	{1230., 1396.}	4.18336	0.0179162	Reff	0.610687	0.618762	{0.617706, 0.619825}	1.32235	ar	63119.	62979.1	{62751., 63200.}	0.292273
20	R0	{1.54435, 5.32911, 0.1}	{1.54465, 5.30296, 4.}	{{1.54465, 5.30296, 4.}, {1.54465, 5.30296, 4.}}	{0.0194651, 0.490783, 3900.}	inf	1762.	1645.34	{1561.32, 1723.51}	6.64954	0.0301869	newly	1849.	1714.22	{1605., 1797.}	7.36939	0.0336141	Reff	0.632813	0.634738	{0.633516, 0.636029}	0.305067	ar	61870.	61521.4	{61278., 61770.}	0.576014
21	R0	{1.57932, 4.90031, 1.}	{1.59034, 5.09935, 4.}	{{1.59034, 5.09935, 4.}, {1.59034, 5.09935, 4.}}	{0.697942, 4.06175, 300.}	inf	1650.	1789.01	{1718.35, 1867.09}	8.42458	0.0349056	newly	1742.	1894.62	{1810., 1981.}	8.76923	0.0362355	Reff	0.608496	0.610532	{0.609272, 0.611601}	0.335094	ar	63595.	64165.4	{63924., 64417.}	0.896863
22	R0	{1.47044, 4.07694, 1.}	{1.4692, 3.88769, 0.}	{{1.44762, 3.83841, 0.}, {1.47608, 4.00571, 0.}}	{0.660777, 4.64202, 100.}	inf	1362.	1272.98	{1171.2, 1377.97}	7.50557	0.0343922	newly	1880.	1747.77	{1580., 1898.}	7.78564	0.0359067	Reff	0.691357	0.692085	{0.681523, 0.696726}	0.784154	ar	55895.	55513.2	{55200., 55849.}	0.709348
23	R0	{1.64997, 3.34546, 0.1}	{1.65456, 3.37285, 0.}	{{1.65456, 3.37285, 0.}, {1.65456, 3.37285, 0.}}	{0.278209, 0.818631, 100.}	inf	230.	242.688	{193.399, 286.813}	14.1851	0.0593411	newly	308.	327.96	{253., 392.}	14.7597	0.0610255	Reff	0.558729	0.560074	{0.559328, 0.560875}	0.241116	ar	66404.	66451.1	{66322., 66562.}	0.126513
24	R0	{1.67405, 4.50254, 0.1}	{1.64953, 4.30394, 2.}	{{1.64953, 4.30394, 2.}, {1.64953, 4.30394, 2.}}	{1.46459, 4.41077, 1900.}	inf	1189.	1009.52	{926.182, 1074.42}	15.0952	0.0717876	newly	1389.	1163.35	{1056., 1247.}	16.2455	0.0778184	Reff	0.560623	0.555372	{0.554116, 0.556567}	0.936497	ar	68017.	67545.1	{67324., 67730.}	0.693753
25	R0	{1.50663, 3.1286, 0.1}	{1.4804, 3.12918, 2.}	{{1.4804, 3.12918, 2.}, {1.4804, 3.12918, 2.}}	{1.74114, 0.0185167, 1900.}	inf	450.	467.212	{389.262, 542.26}	10.7583	0.0455487	newly	698.	704.88	{580., 821.}	10.6218	0.0463428	Reff	0.634201	0.622904	{0.621792, 0.624056}	1.78132	ar	58823.	58673.1	{58424., 58884.}	0.322391
26	R0	{1.69822, 6.34992, 0.1}	{1.69756, 6.15907, 2.}	{{1.69756, 6.15907, 2.}, {1.69756, 6.15907, 2.}}	{0.038572, 3.00556, 1900.}	inf	2723.	2241.2	{2178.46, 2304.42}	17.6936	0.0846845	newly	2372.	2013.42	{1943., 2081.}	15.1172	0.0713509	Reff	0.56493	0.572891	{0.571708, 0.573951}	1.40918	ar	69816.	68975.2	{68770., 69172.}	1.20438
27	R0	{1.55869, 4.92988, 0.1}	{1.55618, 4.83753, 3.}	{{1.55618, 4.83753, 3.}, {1.55618, 4.83753, 3.}}	{0.161327, 1.8733, 2900.}	inf	1548.	1458.73	{1379.45, 1527.59}	5.978	0.0270696	newly	1681.	1624.6	{1530., 1702.}	4.2094	0.0188523	Reff	0.625643	0.626023	{0.624847, 0.627231}	0.130402	ar	62344.	62029.1	{61802., 62266.}	0.515238
28	R0	{1.46085, 6.06899, 0.1}	{1.454, 5.65591, 3.}	{{1.454, 5.65591, 3.}, {1.454, 5.65591, 3.}}	{0.468763, 6.80637, 2900.}	inf	2116.	1924.68	{1830.93, 2010.17}	9.04139	0.0414754	newly	2108.	2008.	{1882., 2120.}	5.04364	0.0227576	Reff	0.722157	0.721553	{0.720309, 0.722873}	0.143455	ar	55484.	54683.	{54283., 55103.}	1.45287
29	R0	{1.63017, 6.25164, 0.1}	{1.67405, 7.28757, 1.}	{{1.67405, 7.28757, 1.}, {1.67405, 7.28757, 1.}}	{2.69172, 16.5706, 900.}	inf	2357.	3175.05	{3092.79, 3253.68}	34.7071	0.129301	newly	2132.	2532.67	{2470., 2606.}	18.7932	0.0746713	Reff	0.606896	0.609537	{0.608166, 0.6109}	0.435231	ar	66231.	67839.5	{67600., 68056.}	2.42859
30	R0	{1.45173, 5.68165, 0.1}	{1.454, 5.65591, 3.}	{{1.454, 5.65591, 3.}, {1.454, 5.65591, 3.}}	{0.156508, 0.452997, 2900.}	inf	2119.	2177.36	{2076.77, 2273.42}	3.5055	0.0148867	newly	2202.	2267.	{2151., 2373.}	3.93188	0.016651	Reff	0.721074	0.721354	{0.719949, 0.722796}	0.115803	ar	55280.	55127.2	{54695., 55536.}	0.529468
31	R0	{1.55808, 2.77316, 0.1}	{1.51531, 2.72346, 5.}	{{1.51531, 2.72346, 5.}, {1.51531, 2.72346, 5.}}	{2.74532, 1.79201, 4900.}	inf	226.	180.844	{135.33, 219.749}	21.6748	0.112821	newly	342.	289.13	{213., 359.}	19.2661	0.097896	Reff	0.613478	0.597321	{0.596697, 0.597996}	2.63379	ar	60940.	60847.9	{60722., 60977.}	0.19045
32	R0	{1.56797, 3.90327, 0.1}	{1.53501, 4.02511, 0.}	{{1.53501, 4.02511, 0.}, {1.53501, 4.02511, 0.}}	{2.10217, 3.12138, 100.}	inf	914.	984.335	{906.942, 1055.2}	8.22152	0.0338078	newly	1187.	1229.03	{1138., 1324.}	5.73799	0.0241419	Reff	0.61673	0.602685	{0.601468, 0.603852}	2.27723	ar	62228.	62164.6	{61932., 62378.}	0.24309
33	R0	{1.70027, 5.43261, 0.1}	{1.65865, 5.3704, 5.}	{{1.65865, 5.3704, 5.}, {1.65865, 5.3704, 5.}}	{2.44779, 1.14517, 4900.}	inf	1215.	1127.99	{1073.55, 1183.18}	7.20231	0.0327752	newly	1142.	1107.62	{1047., 1161.}	4.14011	0.0185043	Reff	0.555682	0.543523	{0.542558, 0.544404}	2.18808	ar	68798.	68512.6	{68338., 68680.}	0.417774
34	R0	{1.61668, 6.86192, 0.1}	{1.57983, 5.14618, 4.}	{{1.54465, 5.09935, 4.}, {1.59034, 5.30296, 4.}}	{2.27914, 25.0038, 3900.}	inf	2934.	1714.31	{1590.52, 1832.54}	41.571	0.233981	newly	2525.	1849.21	{1670., 1995.}	26.764	0.136268	Reff	0.646575	0.651097	{0.638101, 0.656277}	1.29567	ar	64693.	61662.3	{61313., 62002.}	4.68466
35	R0	{1.55955, 3.45071, 0.1}	{1.54098, 3.31039, 5.}	{{1.54098, 3.31039, 5.}, {1.54098, 3.31039, 5.}}	{1.19078, 4.06641, 4900.}	inf	514.	501.133	{437.733, 563.444}	8.23768	0.037049	newly	674.	714.2	{628., 799.}	9.53412	0.039774	Reff	0.608474	0.601427	{0.600444, 0.602388}	1.15819	ar	61783.	61698.5	{61530., 61874.}	0.211952
36	R0	{1.65068, 4.91307, 0.1}	{1.61691, 4.41442, 4.}	{{1.61691, 4.41442, 4.}, {1.61691, 4.41442, 4.}}	{2.04584, 10.1495, 3900.}	inf	1866.	1511.42	{1421.69, 1588.99}	19.0022	0.0918955	newly	2044.	1755.	{1655., 1849.}	14.1389	0.0666253	Reff	0.596341	0.589874	{0.588617, 0.591292}	1.08444	ar	66453.	65560.3	{65345., 65813.}	1.34343
37	R0	{1.46131, 3.06714, 0.1}	{1.4804, 3.12918, 2.}	{{1.4804, 3.12918, 2.}, {1.4804, 3.12918, 2.}}	{1.30619, 2.02271, 1900.}	inf	344.	369.976	{288.808, 433.164}	14.8285	0.0613404	newly	544.	571.83	{444., 671.}	13.6379	0.0575735	Reff	0.646659	0.654721	{0.653783, 0.655911}	1.24672	ar	56452.	56442.	{56217., 56650.}	0.250638
38	R0	{1.71034, 5.2546, 0.1}	{1.737, 5.43578, 0.}	{{1.737, 5.43578, 0.}, {1.737, 5.43578, 0.}}	{1.55867, 3.44798, 100.}	inf	1519.	1756.3	{1685.62, 1826.57}	15.6222	0.0628251	newly	1497.	1713.93	{1626., 1787.}	14.491	0.058509	Reff	0.547719	0.552134	{0.550886, 0.553277}	0.806109	ar	69816.	70227.3	{70048., 70447.}	0.589063
39	R0	{1.43565, 5.57989, 1.}	{1.42137, 5.14098, 3.56}	{{1.41571, 4.94504, 2.}, {1.42659, 5.32185, 5.}}	{0.994684, 7.86598, 256.}	inf	1796.	1525.88	{1353.79, 1699.62}	15.0402	0.0722682	newly	1875.	1703.13	{1542., 1869.}	9.68693	0.04498	Reff	0.709096	0.705877	{0.703567, 0.70799}	0.454021	ar	54407.	53609.6	{52996., 54112.}	1.47547
40	R0	{1.40327, 4.00764, 0.1}	{1.40116, 4.24222, 4.}	{{1.40116, 4.24222, 4.}, {1.40116, 4.24222, 4.}}	{0.150217, 5.85334, 3900.}	inf	1177.	1228.02	{1140.22, 1313.39}	5.82239	0.0244472	newly	1644.	1612.39	{1488., 1731.}	4.99696	0.0221653	Reff	0.717576	0.715783	{0.714556, 0.716995}	0.254149	ar	51976.	51727.7	{51368., 52004.}	0.548484
41	R0	{1.63612, 4.26741, 0.1}	{1.61691, 4.41442, 4.}	{{1.61691, 4.41442, 4.}, {1.61691, 4.41442, 4.}}	{1.17414, 3.4449, 3900.}	inf	805.	851.055	{788.584, 918.948}	6.92652	0.0287722	newly	987.	984.53	{897., 1067.}	5.231	0.0227553	Reff	0.590394	0.582717	{0.581583, 0.58372}	1.30027	ar	65029.	65101.1	{64915., 65292.}	0.191776
42	R0	{1.57426, 6.50271, 0.1}	{1.54465, 5.30296, 4.}	{{1.54465, 5.30296, 4.}, {1.54465, 5.30296, 4.}}	{1.88085, 18.4501, 3900.}	inf	2814.	1968.34	{1888.88, 2066.34}	30.052	0.155479	newly	2604.	2091.26	{1979., 2194.}	19.6905	0.095578	Reff	0.666321	0.666851	{0.665337, 0.668065}	0.144635	ar	62522.	60341.4	{59998., 60678.}	3.48775
43	R0	{1.37818, 5.37636, 0.1}	{1.3618, 4.98002, 1.}	{{1.3618, 4.98002, 1.}, {1.3618, 4.98002, 1.}}	{1.18829, 7.37196, 900.}	inf	1811.	1571.95	{1467.76, 1663.65}	13.1996	0.0619425	newly	2047.	1856.3	{1750., 1978.}	9.31607	0.0429741	Reff	0.756841	0.751103	{0.749805, 0.752359}	0.75817	ar	50098.	48930.8	{48500., 49395.}	2.32993
44	R0	{1.66606, 4.92641, 0.1}	{1.64953, 4.30394, 2.}	{{1.64953, 4.30394, 2.}, {1.64953, 4.30394, 2.}}	{0.99204, 12.6353, 1900.}	inf	1739.	1288.22	{1201.75, 1367.95}	25.9217	0.130829	newly	1807.	1515.43	{1405., 1608.}	16.1356	0.0769753	Reff	0.583371	0.585019	{0.583695, 0.586432}	0.286881	ar	67066.	66217.1	{65953., 66433.}	1.26575
45	R0	{1.623, 5.9449, 0.1}	{1.59034, 5.09935, 4.}	{{1.59034, 5.09935, 4.}, {1.59034, 5.09935, 4.}}	{2.01215, 14.2231, 3900.}	inf	2642.	2042.86	{1944.7, 2149.36}	22.6775	0.111986	newly	2549.	2196.16	{2086., 2309.}	13.8423	0.0650197	Reff	0.641799	0.638414	{0.636704, 0.639945}	0.527519	ar	64554.	63090.3	{62726., 63448.}	2.26734
46	R0	{1.65235, 5.51234, 0.1}	{1.65865, 5.3704, 5.}	{{1.65865, 5.3704, 5.}, {1.65865, 5.3704, 5.}}	{0.381332, 2.575, 4900.}	inf	2356.	2348.28	{2249.01, 2447.7}	2.63668	0.0114818	newly	2372.	2385.05	{2285., 2480.}	2.72976	0.0117806	Reff	0.608825	0.611275	{0.609444, 0.612827}	0.412826	ar	66509.	66412.4	{66111., 66689.}	0.292036
47	R0	{1.62138, 8.82208, 0.1}	{1.76659, 10.896, 5.}	{{1.76659, 10.896, 5.}, {1.76659, 10.896, 5.}}	{8.9561, 23.5088, 4900.}	inf	4208.	5665.76	{5584.1, 5749.71}	34.6427	0.129157	newly	2927.	3296.44	{3209., 3359.}	12.6218	0.0515591	Reff	0.666095	0.699999	{0.698463, 0.70142}	5.08987	ar	65598.	69463.5	{69124., 69795.}	5.89274
48	R0	{1.64073, 6.69261, 0.1}	{1.67405, 7.28757, 1.}	{{1.67405, 7.28757, 1.}, {1.67405, 7.28757, 1.}}	{2.03078, 8.88987, 900.}	inf	3295.	4030.22	{3936.27, 4114.57}	22.3133	0.0873966	newly	2847.	3238.22	{3147., 3328.}	13.7415	0.0558202	Reff	0.626628	0.627045	{0.625546, 0.628601}	0.169267	ar	66438.	68078.2	{67814., 68323.}	2.4688
49	R0	{1.65556, 3.796, 0.1}	{1.65456, 3.37285, 0.}	{{1.65456, 3.37285, 0.}, {1.65456, 3.37285, 0.}}	{0.0603803, 11.1473, 100.}	inf	539.	454.683	{391.737, 527.126}	15.7969	0.0770663	newly	685.	619.67	{536., 716.}	11.3679	0.0538452	Reff	0.564513	0.565567	{0.56434, 0.566481}	0.20014	ar	66575.	66394.9	{66223., 66575.}	0.290529
50	R0	{1.56828, 3.65713, 0.1}	{1.56331, 3.72349, 2.}	{{1.56331, 3.72349, 2.}, {1.56331, 3.72349, 2.}}	{0.31709, 1.81443, 1900.}	inf	506.	523.079	{452.852, 582.798}	8.4245	0.0357056	newly	685.	685.98	{592., 762.}	7.78978	0.0340538	Reff	0.595868	0.593712	{0.592766, 0.594771}	0.361898	ar	62761.	62775.7	{62589., 62929.}	0.17138
